# Precarious Balance: Methylmercury, Selenium, and Cardiovascular Health

**DOI:** 10.1289/ehp.119-a354a

**Published:** 2011-08-01

**Authors:** Naomi Lubick

**Affiliations:** Naomi Lubick is a freelance science writer based in Stockholm, Sweden, and Folsom, CA. She has written for *Environmental Science & Technology*, *Nature*, and *Earth*.

Inuit people living in Canada’s Nunavik region consume large doses of methylmercury through their traditional diet, mostly from sea mammal meat. A new study set out looking for evidence of links between these native communities’ exposure to the toxic metal and heart diseases [***EHP* 119(8):1077–1083; Ayotte et al.**]. The results suggested an inhibitory effect of methylmercury on paraoxonase 1 (PON1), an enzyme whose activity may protect against cardiovascular problems. They also hinted unexpectedly that dietary nutrients, especially selenium, may counteract effects of methylmercury on PON1 activity. PON1 breaks down the oxidized lipids that may promote atherosclerosis.

The researchers collected blood plasma samples from nearly 900 people living in about 500 households in different communities across Nunavik. Each person provided biological samples and completed surveys on their medical history, diet, and lifestyle habits.

A simple correlation analysis indicated that higher blood mercury was associated with higher PON1 activity, contrary to findings from earlier *in vitro* studies. So the researchers turned to multivariate statistics to untangle other factors that might confound the mercury/PON1 relationship. These include intake of omega-3 polyunsaturated fatty acids (n-3 PUFAs) and selenium, important nutrients provided by the traditional Inuit diet.

**Figure:**
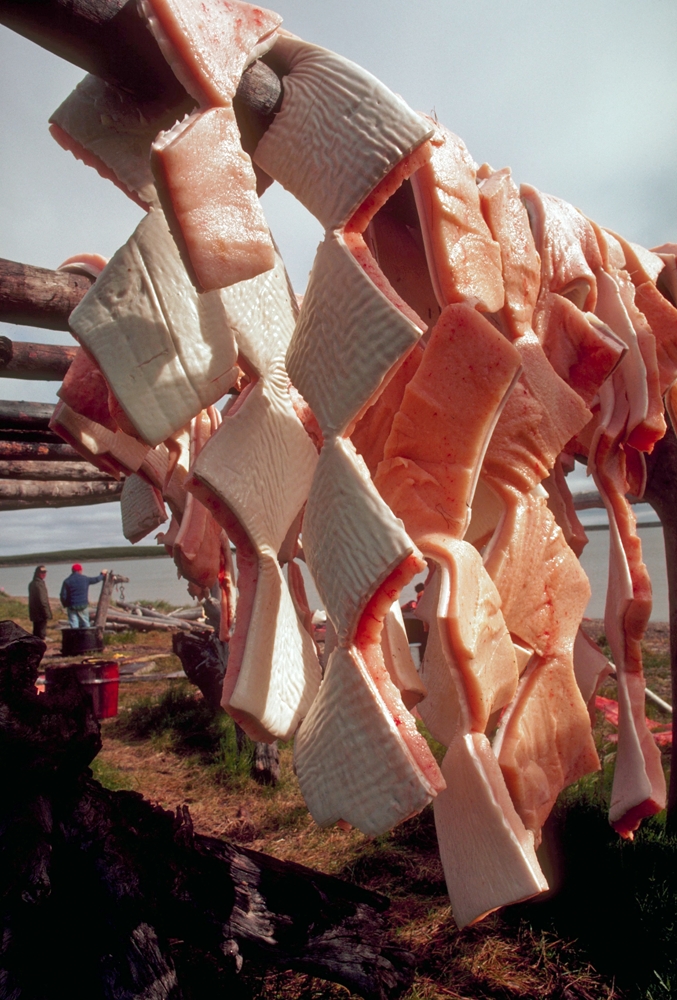
Beluga *muktuk* dries on racks at an Inuit whaling camp. © Lowell Georgia/Corbis

In the multivariate model, higher blood mercury levels were associated with decreased PON1 activity whereas higher blood levels of n-3 PUFAs and selenium were associated with increased PON1 activity. The researchers also observed that PON1 activity decreased with age of participants, as previously reported in other populations.

Fish and sea mammal fats carry n-3 PUFAs that are beneficial for heart health, and have been known for some time to be a key nutrient in the traditional Inuit diet. This diet also includes such high-selenium foods as beluga whale *muktuk* (skin and fat). The researchers suggest that n-3 PUFAs and selenium in the Inuit diet may offset the inhibition of PON1 activity by methylmercury.

But as native populations shift to “modern” diets—for example, eating more fast food and prepackaged meals—the dietary balance between these nutrients and methylmercury could change, the researchers caution. For instance, if an individual consumes less dietary selenium, the negative effects of methylmercury may intensify. The researchers emphasize that followup studies are necessary to monitor dietary changes and cardiovascular health among these Arctic communities.

